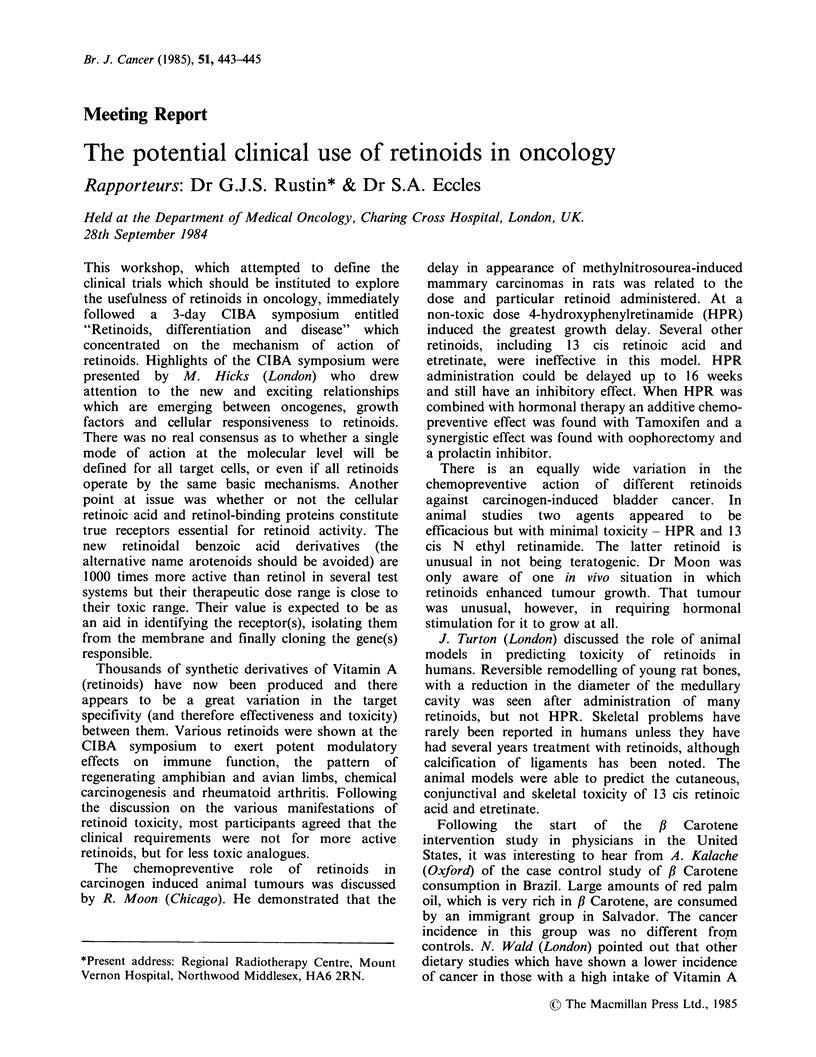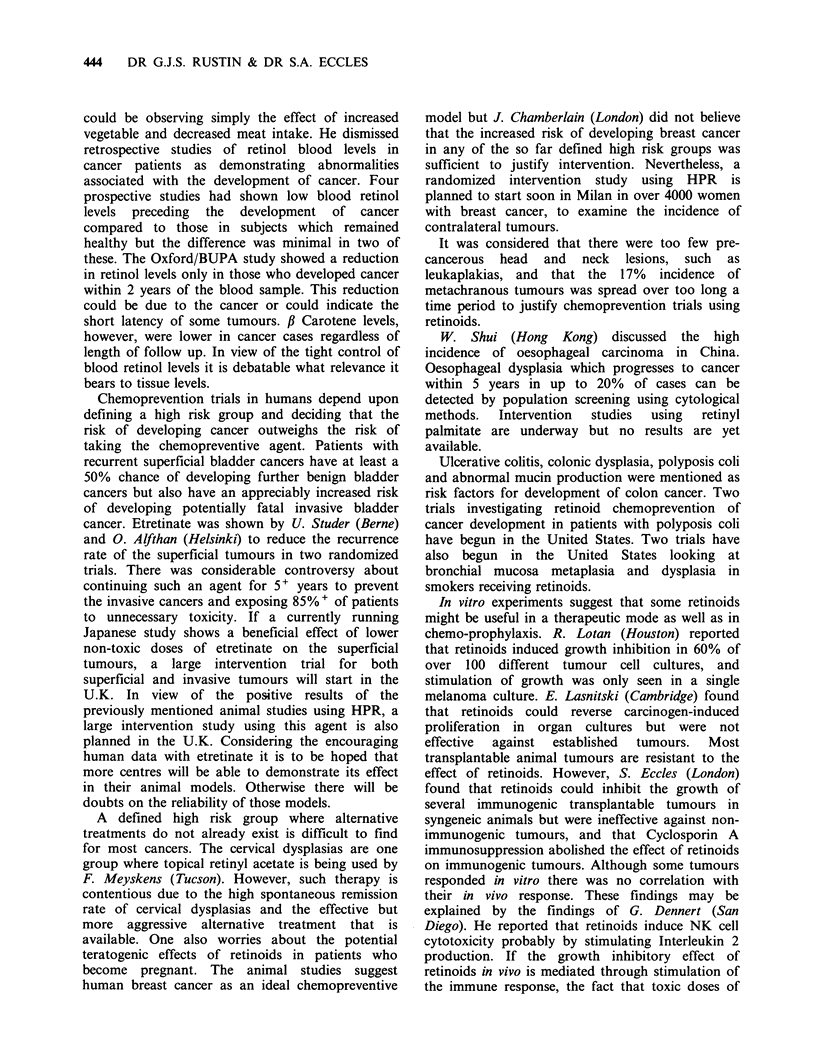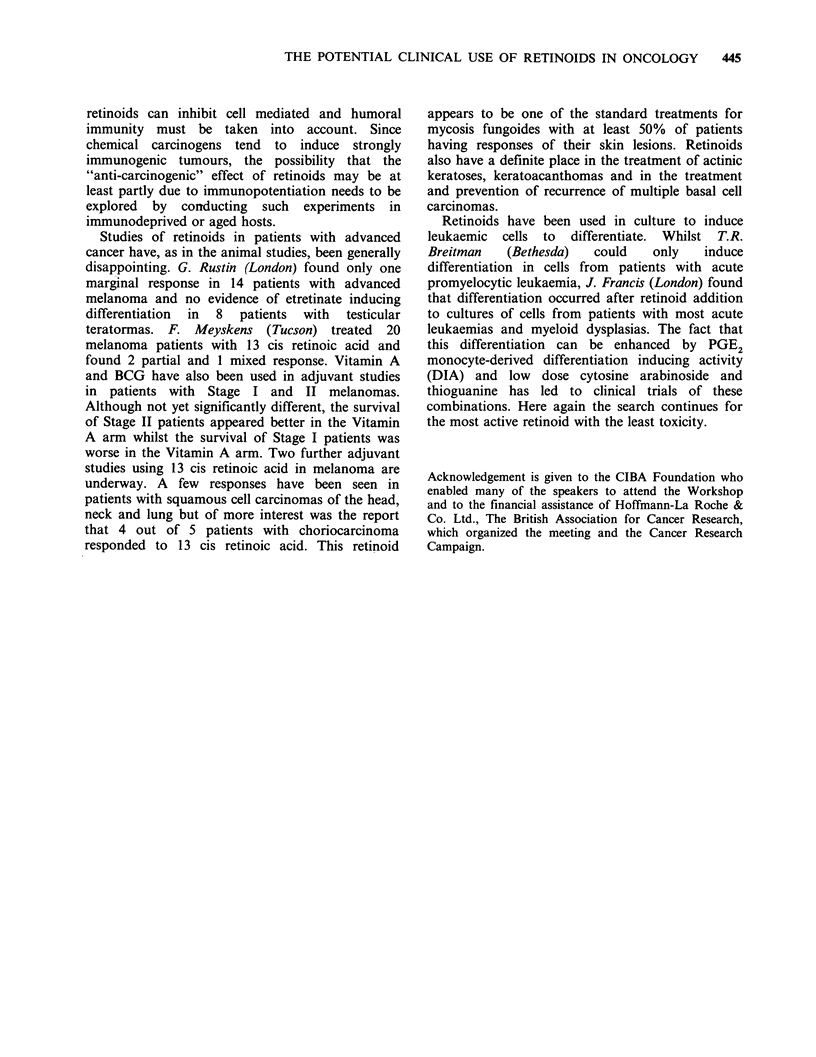# British Association For Cancer Research Workshop on the Potential Clinical Use of Retinoids in Oncology

**Published:** 1985-03

**Authors:** 


*Br. J. Cancer* (1985), **51**, 443–445

### Meeting Report

### The potential clinical use of retinoids in oncology

*Rapporteurs*: Dr G.J.S. Rustin* & Dr S.A. Eccles

*Held at the Department of Medical Oncology, Charing Cross Hospital, London, UK. 28th September 1984*

This workshop, which attempted to define the clinical trials which should be instituted to explore the usefulness of retinoids in oncology, immediately followed a 3-day CIBA symposium entitled "Retinoids, differentiation and disease" which concentrated on the mechanism of action of retinoids. Highlights of the CIBA symposium were presented by *M. Hicks* (*London*) who drew attention to the new and exciting relationships which are emerging between oncogenes, growth factors and cellular responsiveness to retinoids. There was no real consensus as to whether a single mode of action at the molecular level will be defined for all target cells, or even if all retinoids operate by the same basic mechanisms. Another point at issue was whether or not the cellular retinoic acid and retinol-binding proteins constitute true receptors essential for retinoid activity. The new retinoidal benzoic acid derivatives (the alternative name arotenoids should be avoided) are 1000 times more active than retinol in several test systems but their therapeutic dose range is close to their toxic range. Their value is expected to be as an aid in identifying the receptor(s), isolating them from the membrane and finally cloning the gene(s) responsible.

Thousands of synthetic derivatives of Vitamin A (retinoids) have now been produced and there appears to be a great variation in the target specifivity (and therefore effectiveness and toxicity) between them. Various retinoids were shown at the CIBA symposium to exert potent modulatory effects on immune function, the pattern of regenerating amphibian and avian limbs, chemical carcinogenesis and rheumatoid arthritis. Following the discussion on the various manifestations of retinoid toxicity, most participants agreed that the clinical requirements were not for more active retinoids, but for less toxic analogues.

The chemopreventive role of retinoids in carcinogen induced animal tumours was discussed by *R. Moon* (*Chicago*). He demonstrated that the delay in appearance of methylnitrosourea-induced mammary carcinomas in rats was related to the dose and particular retinoid administered. At a non-toxic dose 4-hydroxyphenylretinamide (HPR) induced the greatest growth delay. Several other retinoids, including 13 cis retinoic acid and etretinate, were ineffective in this model. HPR administration could be delayed up to 16 weeks and still have an inhibitory effect. When HPR was combined with hormonal therapy an additive chemopreventive effect was found with Tamoxifen and a synergistic effect was found with oophorectomy and a prolactin inhibitor.

There is an equally wide variation in the chemopreventive action of different retinoids against carcinogen-induced bladder cancer. In animal studies two agents appeared to be efficacious but with minimal toxicity – HPR and 13 cis N ethyl retinamide. The latter retinoid is unusual in not being teratogenic. Dr Moon was only aware of one *in vivo* situation in which retinoids enhanced tumour growth. That tumour was unusual, however, in requiring hormonal stimulation for it to grow at all.

*J. Turton* (*London*) discussed the role of animal models in predicting toxicity of retinoids in humans. Reversible remodelling of young rat bones, with a reduction in the diameter of the medullary cavity was seen after administration of many retinoids, but not HPR. Skeletal problems have rarely been reported in humans unless they have had several years treatment with retinoids, although calcification of ligaments has been noted. The animal models were able to predict the cutaneous, conjunctival and skeletal toxicity of 13 cis retinoic acid and etretinate.

Following the start of the $\beta$ Carotene intervention study in physicians in the United States, it was interesting to hear from *A. Kalache* (*Oxford*) of the case control study of $\beta$ Carotene consumption in Brazil. Large amounts of red palm oil, which is very rich in $\beta$ Carotene, are consumed by an immigrant group in Salvador. The cancer incidence in this group was no different from controls. *N. Wald* (*London*) pointed out that other dietary studies which have shown a lower incidence of cancer in those with a high intake of Vitamin A

*Present address: Regional Radiotherapy Centre, Mount Vernon Hospital, Northwood Middlesex, HA6 2RN.

© The Macmillan Press Ltd., 1985

could be observing simply the effect of increased vegetable and decreased meat intake. He dismissed retrospective studies of retinol blood levels in cancer patients as demonstrating abnormalities associated with the development of cancer. Four prospective studies had shown low blood retinol levels preceding the development of cancer compared to those in subjects which remained healthy but the difference was minimal in two of these. The Oxford/BUPA study showed a reduction in retinol levels only in those who developed cancer within 2 years of the blood sample. This reduction could be due to the cancer or could indicate the short latency of some tumours. β Carotene levels, however, were lower in cancer cases regardless of length of follow up. In view of the tight control of blood retinol levels it is debatable what relevance it bears to tissue levels.

Chemoprevention trials in humans depend upon defining a high risk group and deciding that the risk of developing cancer outweighs the risk of taking the chemopreventive agent. Patients with recurrent superficial bladder cancers have at least a 50% chance of developing further benign bladder cancers but also have an appreciably increased risk of developing potentially fatal invasive bladder cancer. Etretinate was shown by *U. Studer* (*Berne*) and *O. Alfthan* (*Helsinki*) to reduce the recurrence rate of the superficial tumours in two randomized trials. There was considerable controversy about continuing such an agent for $5^+$ years to prevent the invasive cancers and exposing $85\%^+$ of patients to unnecessary toxicity. If a currently running Japanese study shows a beneficial effect of lower non-toxic doses of etretinate on the superficial tumours, a large intervention trial for both superficial and invasive tumours will start in the U.K. In view of the positive results of the previously mentioned animal studies using HPR, a large intervention study using this agent is also planned in the U.K. Considering the encouraging human data with etretinate it is to be hoped that more centres will be able to demonstrate its effect in their animal models. Otherwise there will be doubts on the reliability of those models.

A defined high risk group where alternative treatments do not already exist is difficult to find for most cancers. The cervical dysplasias are one group where topical retinyl acetate is being used by *F. Meyskens* (*Tucson*). However, such therapy is contentious due to the high spontaneous remission rate of cervical dysplasias and the effective but more aggressive alternative treatment that is available. One also worries about the potential teratogenic effects of retinoids in patients who become pregnant. The animal studies suggest human breast cancer as an ideal chemopreventive model but *J. Chamberlain* (*London*) did not believe that the increased risk of developing breast cancer in any of the so far defined high risk groups was sufficient to justify intervention. Nevertheless, a randomized intervention study using HPR is planned to start soon in Milan in over 4000 women with breast cancer, to examine the incidence of contralateral tumours.

It was considered that there were too few pre-cancerous head and neck lesions, such as leukaplakias, and that the 17% incidence of metachranous tumours was spread over too long a time period to justify chemoprevention trials using retinoids.

*W. Shui* (*Hong Kong*) discussed the high incidence of oesophageal carcinoma in China. Oesophageal dysplasia which progresses to cancer within 5 years in up to 20% of cases can be detected by population screening using cytological methods. Intervention studies using retinyl palmitate are underway but no results are yet available.

Ulcerative colitis, colonic dysplasia, polyposis coli and abnormal mucin production were mentioned as risk factors for development of colon cancer. Two trials investigating retinoid chemoprevention of cancer development in patients with polyposis coli have begun in the United States. Two trials have also begun in the United States looking at bronchial mucosa metaplasia and dysplasia in smokers receiving retinoids.

*In vitro* experiments suggest that some retinoids might be useful in a therapeutic mode as well as in chemo-prophylaxis. *R. Lotan* (*Houston*) reported that retinoids induced growth inhibition in 60% of over 100 different tumour cell cultures, and stimulation of growth was only seen in a single melanoma culture. *E. Lasnitski* (*Cambridge*) found that retinoids could reverse carcinogen-induced proliferation in organ cultures but were not effective against established tumours. Most transplantable animal tumours are resistant to the effect of retinoids. However, *S. Eccles* (*London*) found that retinoids could inhibit the growth of several immunogenic transplantable tumours in syngeneic animals but were ineffective against non-immunogenic tumours, and that Cyclosporin A immunosuppression abolished the effect of retinoids on immunogenic tumours. Although some tumours responded *in vitro* there was no correlation with their *in vivo* response. These findings may be explained by the findings of *G. Dennert* (*San Diego*). He reported that retinoids induce NK cell cytotoxicity probably by stimulating Interleukin 2 production. If the growth inhibitory effect of retinoids *in vivo* is mediated through stimulation of the immune response, the fact that toxic doses of retinoids can inhibit cell mediated and humoral immunity must be taken into account. Since chemical carcinogens tend to induce strongly immunogenic tumours, the possibility that the "anti-carcinogenic" effect of retinoids may be at least partly due to immunopotentiation needs to be explored by conducting such experiments in immunodeprived or aged hosts.

Studies of retinoids in patients with advanced cancer have, as in the animal studies, been generally disappointing. *G. Rustin (London)* found only one marginal response in 14 patients with advanced melanoma and no evidence of etretinate inducing differentiation in 8 patients with testicular teratormas. *F. Meyskens* (*Tucson*) treated 20 melanoma patients with 13 cis retinoic acid and found 2 partial and 1 mixed response. Vitamin A and BCG have also been used in adjuvant studies in patients with Stage I and II melanomas. Although not yet significantly different, the survival of Stage II patients appeared better in the Vitamin A arm whilst the survival of Stage I patients was worse in the Vitamin A arm. Two further adjuvant studies using 13 cis retinoic acid in melanoma are underway. A few responses have been seen in patients with squamous cell carcinomas of the head, neck and lung but of more interest was the report that 4 out of 5 patients with choriocarcinoma responded to 13 cis retinoic acid. This retinoid appears to be one of the standard treatments for mycosis fungoides with at least 50% of patients having responses of their skin lesions. Retinoids also have a definite place in the treatment of actinic keratoses, keratoacanthomas and in the treatment and prevention of recurrence of multiple basal cell carcinomas.

Retinoids have been used in culture to induce leukaemic cells to differentiate. Whilst *T.R. Breitman* (*Bethesda*) could only induce differentiation in cells from patients with acute promyelocytic leukaemia, *J. Francis* (*London*) found that differentiation occurred after retinoid addition to cultures of cells from patients with most acute leukaemias and myeloid dysplasias. The fact that this differentiation can be enhanced by $PGE_2$ monocyte-derived differentiation inducing activity (DIA) and low dose cytosine arabinoside and thioguanine has led to clinical trials of these combinations. Here again the search continues for the most active retinoid with the least toxicity.

Acknowledgement is given to the CIBA Foundation who enabled many of the speakers to attend the Workshop and to the financial assistance of Hoffmann-La Roche & Co. Ltd., The British Association for Cancer Research, which organized the meeting and the Cancer Research Campaign.